# The identity changes in online learning and teaching: instructors, learners, and learning management systems

**DOI:** 10.1186/s41239-021-00304-8

**Published:** 2021-12-28

**Authors:** Soyeong Kwon, Woolchul Kim, Changyeon Bae, Minjang Cho, Seunghoon Lee, Neal Dreamson

**Affiliations:** 1 Stony Brook University, Stony Brook, USA; 2 Department of Technology & Society, The State University of New York, Korea (SUNY Korea), Incheon, Korea

**Keywords:** Online learning, Instructor identity, Learner identity, Learning environment, Learning management systems (LMS)

## Abstract

Not because of the unexpected global pandemic, but because of the emergence of educational technology and pedagogical innovation, the ways of teaching and learning have been switched to technology integrated modes such as blended and flipped learning which is more than changing to online from face-to-face. Yet, many institutes, which rely on a conventional residential teaching mode or use learning management systems (LMS) as an additive tool, are further struggling to adjust to the new environment. In this paper, we argue that the identity changes of three components, instructor, learner, and LMS are inevitable for authentic online teaching and learning. By applying conceptual frameworks for the identity changes with four sequential levels, we evaluated Blackboard course sites (n = 53) and analysed course evaluations (n = 41) from a university that remained holding a traditional classroom mode and using an LMS in a non-integrated way. As a result, only a few courses appeared at higher levels of the identity changes. To integrate the identity changes in online learning and teaching, we argue that an LMS should be designed and managed as a learning community; both instructors and learners should be repositioned as co-participants; and they should work together to build a post-learning community by practicing community membership.

## Introduction

It has been a long time since technology integrated teaching and learning was adopted in higher education—In fact, the word ‘e-learning’ was coined in 1999, and most major universities in Western countries have used a Learning Management System (LMS) as part of their formal educational activities since the late 1990s and developed blended and complete online programs (Cross, 2004; Pishva et al., 2010). Yet, online teaching and learning has been (re-)spotlighted due to the COVID-19 pandemic, and its impact is on the way campuses operate and how stakeholders communicate with one another. In particular, online learning and teaching have been challenging those universities which maintain a classroom-based teaching model while an LMS is used as additive (Dreamson, 2020; Dhawan, 2020; Picciano, 2017) and instructors and learners who are either unfamiliar with or yet unaware of pedagogical values of digitally networked learning environments (Dhawan, 2020; Dreamson, 2020; Picciano, 2017). On the surface, technology integrated education requires some basic technical skills such as presentation slides with voice-over, video recorded lecturing, and live streaming lecturing, which becomes mandatory, as observed today. However, it is often ignored that the pedagogical values require more than getting familiar with those technical skills, and they are inclusive of value of care, value of diversity, value of community and value of justice (Palahicky, et al., 2019). More fundamentally, the values require ‘identity changes’ including instructor, learners, and the learning environment, as technology offers a new paradigm of education (e.g., an instructor to a learning designer, an instructor to a learning partner, and a knowledge transmitter to a co-knowledge constructor) (Conrad & Donaldson, 2012; Khalid, 2019; Park, 2011; Parra, 2013; Salmon, 2013).

The changes could develop great resistance which also results in tokenistic professionalism at best which is focused on functional convenience: Using an LMS as a digital depository and/or a bulletin board, offering self-learning activities, structuring a course based on one-to-many (an instructor-many individual students) interactions, and/or the absence of teacher presence in communication and collaboration (Dreamson, 2020; Brown et al., 2019; Park, 2011; Rapanta et al., 2020). These approaches could degenerate the underlying pedagogical strategies such as interactive learning (Anderson et al., 2001), personalised learning (Temdee, 2020), collaborative learning (Harasim, 2000), connectivist learning (Siemens, 2004), and smart learning (Khlaif & Farid, 2018). Inversely, the current hardship, even though it would not be long, offers opportunities for higher education to systematically and thoughtfully consider the identity changes. Great opportunities are that people across diverse sectors have started thinking about making provisions for recurrence of the pandemic and considering an upgrade of the current teaching and learning systems (Dreamson, 2020; Rapanta et al., 2020).

In this pandemic period, probably, those who record their lectures in either voice or video and/or undertake real-time video conferencing, already realised that the biggest challenge they encountered could be the identity changes. For example, it has been known that the authenticity of online education can be found in enhanced ownership and authorship of learning through technology integrated collaborative learning and co-construction of knowledge (Rapanta et al., 2020). It was reported that those learners who are less internally motivated for academic interests in an online learning environment often experience strong negative affections such as boredom, anxiety, sadness and frustration (Felicia, 2015). Furthermore, both instructor identity and learner identity are highly related to their perceptions and ways of using an LMS (also known as a virtual learning environment) (Park, 2011). In essence, an LMS is a technology integrated learning environment where instructors are supposed to have the ability of using it for course design, interaction and collaboration, assessment, and learner support, and thereby, learners are given opportunities to implement their creative and collaborative learning engagement strategies (Khlaif & Farid, 2018; Rapanta et al., 2020; Temdee, 2020).

In this paper, the identity changes of the three components, instructor identity, learner identity, and LMS identity, are explored. First, we review contemporary literature in online learning and teaching to articulate how each identity is addressed. Specifically, we review online learning frameworks to structuralise the identity changes. Second, we articulate key features of each component as a conceptual framework to be used to evaluate whether the identity changes are evidenced in online course sites (n = 53) and course evaluations (n = 41). Third, we identify their perceptions of and experiences in the identity changes and address challenges that prevent the identity changes and thus suggest pedagogical recommendations for effective identity changes.

## Identity changes in online learning and teaching

In online learning and teaching, instructor identity is shifted from “a didactic purveyor of information” to “an interactive instructor” (Park, 2011, p. 179). In their empirical study on instructor identity, Heuer and King (2004) argued that instructors hold multiple identities: *a leader* who demonstrates a role model of active participation, *a coach* who encourages learners to create a team, *a facilitator* who takes responsibility for learner success and engagement, and *a communicator* who fosters communication and collaboration. A similar study is also found in Bonk et al.’s study (2018) that instructors should be engaged in a pedagogical domain for interactive learning and teaching strategies such as providing feedback, facilitating discussion, synthetically analysing student comments, and connecting to outside resources and experts. Yet, they criticised that the strategies often remain unconsidered and unorganised in many online courses because of the unawareness and no application of the identity changes.

Such identity changes of instructors are the same to learners. In essence, learners can have opportunities to develop identities by participating in co-construction of meaning rather than consuming existing knowledge. In a study on learner identity, Khalid (2019) observed that learners’ identity changes occur when an online course is managed and facilitated towards building an online community by ensuring that learners have a shared goal and needs beyond learning objectives. Indeed, shifting learner identity from “a knowledge receiver” to “an interactive learning participant” is the ultimate benefit of online learning (Park, 2011, p. 179). This identity change is derived from the fact that quality learning is achievable through active participation and engagement in online learning environments for the reason: if quality human-to-human interactions are not organised in the online learning environment, technological features and learner needs remain mismatched, and the environment becomes a tool for self-learning (Park, 2011).

In their comparative experimental study on social presence through interactions between instructor and students in a course for 8 semesters, d’Alessio et al. (2019) discovered that higher frequent announcements and feedback strengthen student–student and instructor-student connections, which, in turn, further enhances the instructor’s pedagogical belief that is to build a supportive community. In the interconnected socio-emotional aspects of online learning, it is argued that an LMS can become a social space when consistent connections between interaction and sense of community are facilitated, which not only increases knowledge co-construction but also encourages learners to be aware of their emergent identities (Delahunty et al., 2014). In fact, Online learning is not only a full version of self-paced learning but also promotes diverse learning modes such as participatory, collaborative and representative modes (Dreamson, 2020). In this sense, an LMS becomes a learning space where a number of built-in communication and collaboration tools such as blogs, wikis, forums, groups, journals, and conferences are available, and these tools remain unused in many cases or the usages happen at the beginning of courses only due to ill-defined participation, poorly designed communication channels, and learning contents formatted for downloading only (Dreamson, 2019; Rapanta et al., 2020).

## Frameworks for online learning and teaching

Such identity changes need to be systematically structured to find evaluate online courses, and the following three frameworks directly address the changes in higher education contexts: Conrad and Donaldson’s (2012) *phases of engagement*, Salmon’s (2013) *e-tivities*, and Parra’s (2013) *phases, scaffolds, and technology for collaboration*. All these frameworks are to suggest sequentially structured four or five stages/phases of an online course that require different roles of instructor and learner and different ways of using the LMSs including embedded and external technologies.

### Phases of engagement

Conrad and Donaldson (2012) structured five phases: connect, communicate, collaborate, co-facilitate, and continue for quality online learning and teaching. They argued that both instructor and learner identities are transformed at each phase. In the first *connect* phase, instructors are *a social negotiator* for newcomers who ensures that learners are aware of learning expectations and ready to be active in learning. The instructor is supposed to offer activities to encourage learners to be interactive with peer learners, and thus, the LMS needs to be designed to facilitate communications for learners to not only understand learning requirements but also raise their voice through interactions with peer learners and the instructor. In the second *communicate* phase, learner-to-learner interactions in pairs are initiated and facilitated, where critical thinking, reflection, and sharing of ideas are required, which changes the instructor role to *a structural engineer* who designs the interactions such as peer reviews, activity critiques, and discussions. At this phase, the LMS is transformed into *a learning community* where quality human-to-human interactions occur. In the third *collaborate* phase, the instructor role as *a facilitator* is to support quality collective human-to-human interactions through activities such as discussion, role playing, debates, and jigsaws. In this context, learners are supposed to hold *membership* of a learning community. In the fourth *co-facilitate* phase, instructors and learners become *equal participants*, they hold *a partnership relationship* in which group projects are developed and implemented, and thus, the LMS is fully transformed into a learning community. In the fifth *continue* phase, instructors become *a supporter*, and learners become *a contemplator,* and both are engaged in course evaluation and self-reflection on learning activities. In this phase, transformed identities should be observed if the course is successful.

### E-tivities

In Salmon’s (2013) e-tivities model, a learning environment is understood in a linear structure where learning is sequentially developed through five stages: access and motivation, online socialisation, information exchange, knowledge construction, and development. For Salmon, learning ultimately aims to construct knowledge and apply it to real-life situations, and an LMS is considered a space where active and participatory learning occurs. In this environment, instructors need to be *a facilitator* who can moderate and technically support learners to move to the next stages. She also addressed learners’ identity change as *knowledge constructors* and *collaborators*. In the first stage, *access and motivation*, learners are assured that they get familiar with the LMS and required technological tools, and the instructor needs to organise it for easy access and encourage individual learners to be aware of what they are going to achieve in terms of quality human interactions as well as learning outcomes. In the second stage, *online socialisation*, the LMS becomes a space where messages are exchanged, and the instructor ensures that the gaps between cultural, social and learning environments are bridged, which is necessary for learners to be aware of diverse and potential issues and learning expectations. In the third stage, *information exchange*, learners are engaged with developing artefacts and/or substantially using technologies for communication, collaboration, and creation. Specifically, learners are given a social role to develop rules, expectations, and procedures towards *a constructive and healthy learning community*. In the fourth stage, *knowledge construction*, collaborative learning is realised in ways that learners are highly engaged in interpersonal and intrapersonal communication and hold full *membership* of the LMS as a community. The instructor is supposed to facilitate quality discussion and collaboration in order for learners to build internal representation of knowledge. In the fifth stage, *development*, learners explore their knowledge building process and evaluate their engagement in the course and technology and its impact on learning, whereas the instructor promotes and enhances critical self-reflection. At this last stage, the LMS becomes equivalent to a full learning community where learners practise their ownership and authorship of learning.

### Phases, scaffolds, and technology for collaboration

Parra (2013) suggested a 16-week learning schedule into four phases depending on the main objectives to be achieved: commencing, practicing, conducting, and celebrating. In the progress, (a) instructor identity is transformed into multiple roles such as *a coordinator, master learner, role model, motivator, mediator, and counsellor*; (b) learner identity is determined by relevant learning activities that highlight interaction, communication, and collaboration; and (c) an LMS is designed and used for collaboration and group work. In the first phase, *commencing*, learners are getting started with group work via multiple communication tools to understand activities, review learning resources and practise communication tools. The instructor’s primary roles are to *coordinate* learner engagement and to *motivate* learners to be ready for quality group work. In the second phase, *practicing*, learners practise collaboration skills in group tasks. While the instructor roles continue, the motivator role is replaced with *a master learner role* which is to facilitate group work and role guide for quality collaboration. In the third phase, *conducting*, learners are focussed on group projects through online meetings and artefact development. Collaboration tools embedded in an LMS, and external communication tools are fully used. In this phase, the instructor role is switched to *a counsellor* who ensures that learners undertake group work through participation and involvement, and both instructor and learners attend meetings, review recordings, and discuss work progress. In the last phase, *celebrating*, learners implement group presentations using a web conferencing tool and finalise their projects. The instructor’s *coordinator* role is back at this stage, while they hold coordinator, counsellor, and motivator roles.

## Conceptual frameworks for the identity changes

The three frameworks not only present what things, aspects, and values instructors should prepare, coordinate, intervene, and facilitate in online learning and teaching but also explicitly indicate their identity is transformed at each stage/phase. Their linearly and sequentially structured stages/phases also reflect the changes of learner engagement in learning activities as per the amount of interactivity which continues to increase throughout a course until groups of learners achieve knowledge co-construction, and individual learners attain full membership. Furthermore, the amount of interactivity determines the extent to which an LMS becomes a learning community. Therefore, it is legitimate that such identity changes of instructor, learner, and LMS are considered the barometers of authentic online learning and teaching when each change is synthesised and articulated in a course. Table 1 presents the identity changes which are retrieved from the three frameworks, and thereby, the stages of identity formations are articulated in a 15 weeks course period as an example.

**Table 1:** The identity changes of online learning and teaching

Identity formation	Period	Instructor identity	Learner identity	LMS identity
Pre-membership	1–3 weeks	Designer	Communicator	Communication channel
Semi-membership	4–5 weeks	Facilitator	Participant	Interaction channel
Full membership				
Inward membership	6–12 weeks	Partner/collaborator	Constructor/collaborator	Collaboration space
Outward membership	13–15 or more weeks	Partner/community member	Collaborator/advocator	Community place

The three frameworks indicate that the identity changes of the three components towards knowledge co-construction in a group, which is regarded as being equivalent to a learning community where the changes of membership occur. First, in the beginning of a course, membership remains undefined and inexperienced, and this pre-membership stage is aimed at ensuring that the members are aware of course expectations through communication channels that the instructor predesigned. Second, familiarising with a customised LMS and learning objectives is focused to facilitate learners to build their pre-membership into semi-membership through interactive activities of individuals and groups. Third, when learners are fully engaged in learning through collaborative knowledge construction and inter-group interactions, they become full members of the learning community. Yet, fourth, their membership remains inside the community unless their relationship building moves towards outside it, or outer-group involvement is facilitated to practise their authentic membership. This means that when both inward and outward membership are promoted, a sense of belonging and community can be maximised, which enables learners to advocate learning outcomes against outer communities, which results in authentic ownership and authorship of learning.

Based on such conceptual understandings of the identity changes and formation, online courses can be evaluated as to whether the identity changes occur, and thereby the authenticity of online learning and teaching is assessed. Tables 2, 3, 4 present evaluation indicators of the three identities.

**Table 2:** Evaluation indicators of instructor identity changes

Instructor identity	Indicator
Designer	Communication, interaction, and collaboration channels are structured in the LMS, and the learning objectives towards a learning community are explicit in the course profile
Facilitator	Activities and tasks for individual learners and groups of learners are organised and facilitated through communication tools such as announcements and group pages are managed
Partner/collaborator	The instructor contributes to group activities and interactions by providing comments, feedback, demonstration, and additional resources and participating in projects or problem-solving
Partner/community member	The instructor leads or supports the members to hold ownership and authorship of learning by facilitating inter-group sharing and/or outer community engagement

**Table 3:** Evaluation indicators of learner identity changes

Learner identity	Indicator
Communicator	Learners are engaged in socialisation and understand the learning objectives through communication. They are also given opportunities to express their expectations and/or understandings of activities/projects
Participant	Individual learners and groups (or pairs) of learners are distinguished, and their engagement and participation are evidenced in diverse structured communication and collaboration tools on a regular basis
Constructor/collaborator	Learning outcomes (e.g., artefacts, techniques, arguments, strategies and frameworks) are constructed by learners’ inputs through collaboration partnership
Collaborator/advocator	Learning outcomes are presented, shared, and/or exhibited in a medium(s) where peer engagement and assessment and/or external promotion and engagement with people is evidenced

**Table 4:** Evaluation indicators of LMS identity changes

LMS identity	Indicator
Communication channel	Interpersonal communication channels are organised and introduced (e.g., self-introduction, Q&A, and group forming)
Interaction channel	Activities for individuals and groups are organised, and inter-group connections are available (e.g., real-time document editing and sharing for individuals and groups)
Collaboration space	Regular group meetings, scheduled outcomes through in-group sharing and peer assessment are implemented (e.g., groups produce artefacts and share them with other groups in a workspace)
Community place	Inter-group engagement or external promotion of learning outcomes is organised and facilitated (e.g., groups present their work in an online community and interact with other participants)

## Study design

We collected study samples (i.e., Blackboard course sites and course evaluations) from an American college whose education type was a traditional residential mode and adopted an LMS in a non-integrated way before the COVID-19 outbreak. The college consists of the five departments, and we accessed 53 Blackboard course sites offered in the spring semester 2020 across the departments as follows: Applied Mathematics and Statistics (n = 9), Mechanical Engineering (n = 7), Technology & Society (n = 11), Computer Science (n = 13), Business Management (n = 7), and Social Science and Humanity (n = 6). Of 53, we were able to access 41 course evaluation results where student written feedback was evident. In this qualitative research, we secured the study findings that were reliable and credible which is called trustworthiness (Lincoln & Guba, 1985; McGloin, 2008). In practice, the degree of consistency between concepts (i.e., the identity change frameworks) and the confidence with findings are critical for trustworthiness in our two sets of data collection and analysis: Blackboard course sites and course evaluations (Lincoln & Guba, 1985; McGloin, 2008).

First, Blackboard course sites. We theoretically verified the consistency of the identity changes by building conceptual frameworks through a review of literature (i.e., Tables 2, 3, 4) (Tobin & Begley, 2004). A conceptual framework as an outcome from a systematic review of literature is a tentative theory that reflects specific phenomena (Maxwell, 2012), and it can be used to assess specific contexts (Petticrew & Roberts, 2006). In qualitative research, furthermore, a conceptual framework is used not only to design research questions but also guide data selection and interpretation which also clarifies the underlying influences of the observed phenomena (Reeves et al., 2008). In practice, we evaluated each Blackboard course site against the indicators of each identity change framework. We visited a site and reviewed each page to identify any indicator from the first to the fourth levels of each framework. As a result, we were able to collect evidence of the indicators, as presented in Tables 5, 6, 7 in the next section and counted each site against the four levels of each framework.

**Table 5:** Blackboard use

Usages	N	Percent
No evidence of Blackboard use	12	22.6
Assignment submission & grade only	12	22.6
Announcements, syllabus, weekly learning materials, assessment submission & grade	21	39.7
Communication and collaboration tools (e.g., blogs, journals, wiki, real-time document editing, YouTube, and so on)	8	15.1
Total	53	100.0

**Table 6:** Learner identity

Identity	N	Percent
Viewer (communicator)	48	90.5
Participant	2	3.8
Constructor/collaborator	2	3.8
Collaborator/advocator	1	1.9
Total	53	100.0

**Table 7:** Instructor identity

Identity	N	Percent
Announcer	44	83.0
Coordinator	4	7.5
Facilitator/collaborator	3	5.7
Partner/community member	2	3.8
Total	53	100.0

Second, course evaluations. We analysed student comments on the course evaluations. Of 53 courses, 41 course evaluations were available with student comments. The comments were sorted out and analysed as per the three stages of thematic analysis: descriptive coding, interpretive coding, and overarching themes (King & Horrocks, 2010). In the first stage, we read the comments and coded texts related to the three identities. In the second stage, we coded ‘communication’, ‘interaction’, and ‘collaboration’ for LMS identity, ‘learning’ and ‘teaching’ for instructor identity, and ‘engagement’ for learner identity. In the third stage, we discovered overarching themes that represented their online learning experiences. As a result of the analysis, three themes emerged: ‘perceptions of online learning’, ‘complaints against instructors’, and ‘expectations of online learning’.

## Analysis of Blackboard course sites

In this section, we present the evaluation results of 53 Blackboard course sites based on the identity changes of LMS, learner, and instructor. Most of the course sites remained at the low levels of the identity changes.

As presented in Table 5, four different ways of using Blackboard as per the four levels of the identity changes were identified and classified. Although eight course sites (15.1%) offered communication and collaboration tools, the tools were ‘inactive’ (e.g., a few threads at the beginning of the semester) or had no input in two courses. As a result, only six course sites appeared ‘active’. In addition, while 30 out of 53 (56.6%) used Blackboard for task completion and summative assessment submission, another six courses offered real-time conferencing sessions using either ZOOM or Google Meet and/or a live chat application for individual consultation upon request, oral presentation, class discussion, and/or group tasks. Consequently, communication, interaction, and/or collaboration were evident in 12 courses, and it was noted that the quality of learner engagement varied: two courses used Discussion Board; six courses used real-time conferencing and chatting tools for group activities; and three courses used real-time conferencing and document editing tools for group projects. Against Table 4*Evaluation indicators of LMS identity changes*, overall, the majority of the courses used Blackboard as either a ‘digital depository’ or ‘communication channel’ at the lower levels of the identity formation, whereas the ‘collaboration space’ and ‘community place’ of the higher levels of the identity formation were evidenced in a few courses, which is consistent with the learner identity changes presented in Table 6.

The learner identity appeared mostly consistent with the ways of using communication and collaboration tools, in that they remained ‘viewers’ (90.5%). Although there were a few, ‘participant identity’ was evidenced in some courses where discussion boards were arranged and facilitated; ‘Constructor/collaborator identity’ appeared in some other courses where group projects were instructed and managed through real-time conferencing and real-time document editing tools (e.g., Google Docs and OneNote); and ‘Collaborator/advocator identity’ was observed in one course only where multiple communication and collaboration tools were used to build a digital presence in the networks. Such learner identities were also consistent with the instructor identities, as presented in Table 7.

When the instructors were engaged in communication, interaction, and collaboration, their identity appeared on the Blackboard sites as ‘announcer’ (83%) who informed the updates of learning materials and resources. ‘Coordinator’ was evidenced in the announcements, which informed instructions for tasks as part of summative assessment (e.g., quizzes and oral presentations). By contrast, a few courses showed evidence of the higher levels of the instructor identities. In the courses, learner-to-learner communication, interaction, and collaboration were structured in tools, and the instructors not only provided what individual learners and groups of learners were supposed to do for quality engagement but also participated in the activities. In particular, it was observed that one course promoted and facilitated a sense of learning community by letting groups of learners to produce artefacts and share on a YouTube channel and interact with viewers, and interactions with peer learners and external viewers were part of both summative and formative assessment.

The evaluation indicates that the LMS was mostly used as a digital depository and/or a bulletin board; the learner identity remained passive learners; and the instructor identity mostly appears as an announcer. Against the identity formation levels in Table 1, the majority of the courses remained at the first and second levels — pre- and semi-membership. In other words, the majority of the course sites show that conventional identities of instructors and learners (knowledge transmitter and knowledge receiver) were presumably adopted, and thereby limited or no development of membership was facilitated. On the contrary, those courses which showed the highest level of the identity formation (i.e., full membership) was evidenced by multiple tools organised and facilitated for communication, interaction, and collaboration, and the instructors were co-participants.

## Analysis of course evaluations

The thematic analysis of 41 course evaluations discovered perceptions of online learning, complaints against instructors, and expectations of online learning. The student comments are directly associated with quality communication, interaction, and collaboration, which also indicated the ways that Blackboard should be used, and perspectives that instructors need to reconsider for authentic online teaching and learning.

First, the majority of the learners perceived that online courses were different from conventional ways of learning courses (in-person class). Learners stated, “online class limits students from understanding the class material better” and “This course turned out to be very difficult after it changed to online”.

Second, the majority of the learners also indicated that they experienced difficulties in asking questions in a prompt manner during online classes, communicating with peer learners for group work, and completing examinations. They provided the reasons: “the class with pre-recorded lectures”, “the traditional format of the exam”, “no interaction opportunities using technologies on Blackboard”, “no instruction on how to organize group meetings in the course”, “the ppt files he reads on class”, “shows videos in class”, “does not communicate with students”, “doesn’t reply to student’s email”, “students tend to remain passive by not turning their video camera”, and “hard to ask the instructor questions when we have initiated our group activity”. These reasons can be classified as the three identities: (a) the Blackboard sites and technological tools were not organised for communication, interaction, and collaboration; (b) the instructors were focused on knowledge transmission and acquisition; (c) the learners received no instruction for their active engagement in online tools. For these reasons, they remained inactive and passive.

By contrast, third, their expectations of online learning became clear, which was evidenced in their positive statements for the courses where communication and collaboration tools were used, and the instructors were identified as collaborators and partners. Learners described, “The professor always gave us clear instructions and notifications about the class materials”, “It was easy to follow because the instructor made a group chat for everyone and kept updating us”, “Despite the online situation, it was easier to approach the instructor (short meetings were easily scheduled through Google Meet), feels like the slides contain much more information than offline courses did”, “It was a great opportunity to be more familiar at handling serious tasks like online exams, and become better at using the technology”, “The Blackboard site was very well-organised” and “the professor was active in consulting and communication”, and “[Professor] allowed us to communicate with him … the more feedback we received, the great outcome we get”. These responses indicate that the higher levels of the identity changes are not only valid but also effective for authentic online learning and teaching.

## Identity changes

The data analyses showed that the most courses had none or fewer identity changes: they remained at the early stages of the identity formation; the LMS was mostly used as a digital depository with no or minimal communications; and as a result, it was an inevitable corollary that a lack of interactions between learners and instructors were observed. With these outcomes, we reached three practical questions that should be solved to promote the identity changes: (a) How to ensure that an LMS is used as a learning community other than a digital depository; (b) How to deal with minimal and unstructured communication, interaction, and collaboration between learners and instructors/learners and learners that hinder instructor identity changes; and (c) How to support both instructors and learners to engage with the identity changes in a teaching and learning process?

### Post-learning community

While an LMS can be used for storing learning resources and materials, its fundamental identity needs to be regarded as an online learning community in a pedagogical sense. It is more than a software application for teaching and learning administration because it operates in the digitally networked environment rather than as a standalone, and a course itself is a (pre-)community, as the stakeholders share learning objectives. In their study for LMS-based evaluation to determine academic efficiency performance, Santiago et al. (2020) argued that the configuration of such a virtual classroom should be designed for quality interactions that provide “support for teaching through virtual forums, encourage teaching innovation, promote communication between different users, facilitate student tracking, self-learning, and self-evaluation, and provide teaching experiences mixed with varying degrees of virtuality” (p. 4). In this understanding, both instructors and learners are required to participate in their learning community (a course per se is a pre-community) towards building a new or post-learning community as a node in the digitally networked environment (Siemens, 2004; Dreamson, 2020).

### Co-participants

As observed from the data analysis, minimal and unstructured communication, interaction, and collaboration were the primary reason why identity changes rarely occurred. In the course sites, the instructors appeared as authoritative figures whose primary role was to transmit knowledge and skills to ‘students’, where the identity changes were not considered, as they were perceived as ‘fixed’. In a study on teacher identity changes in e-learning, Aboud (2020) argued that the instructor identity changes are ‘natural’ because of technological impacts on their personal, professional, situational, and contextual engagement — an LMS is a network node. Specifically, in their empirical study on online teacher identity, Richard and Alsup (2015) addressed “control over course design and teaching, attention to projections of teaching persona or presence, developing and maintaining social aspects of the course, structure and planning, and effective communication with students” that characterise the identity of online teachers (pp. 143–144). This means, both instructors and learners need to be aware that their fundamental identity in online learning and teaching is an active co-participant, and their quality and regular interactions should be scheduled and implemented with diverse identities.

### Community membership

Although learners are expected to be active participants, collaborative knowledge constructors, and learning community members, they remain passive not only because of non- or ill-structured learning activities and LMS but also because of their uncritical perception of a hierarchical teacher-student relationship (Dreamson et al., 2017). This means that learners need to be not only instructed to participate in an online course but also trained for quality online learning. In a study on itinerant online postgraduate learners, Koole (2014) demonstrated that online learners have the capacity to “employ a variety of strategies in interpreting and enacting their identities” and “manage their identity performances and strategies for ontological re-alignment (reconceptualisation of oneself)” (p. 52). Reversely, this means, if a course is managed in ways that the instructor delivers lectures, which is one-way, learners will remain ‘actively passive learners’, as they used to be. That is, re-construction of online courses where more active participation of learners is required should be prioritised.

## Why resistant to identity changes

In the courses where the high levels of identity changes were observed, common methods were that (a) communication and collaboration tools were organised and facilitated for both individual learners and groups of learners, (b) learners were encouraged to participate in activities and instructed to make contributions to group achievements on a regular basis, and (c) the instructors were also co-participants. Then why do such high levels of identity changes rarely occur? As Sun et al. (2018) pointed out, community for online learners has been rarely studied in a comprehensive way because of teaching and learning dichotomies that prevent us from being engaged with ‘reality’. Researchers argue that the fundamental reason can be found in a didactical system that supports conventional or dichotomous teaching and learning practices (Albano, 2017; Chevallard, 1985; Schoenfeld, 2012; Wozniak et al., 2016). Although the following didactical system was discussed in mathematics education, their foundational questions towards the identities of teachers, learners, and learning environments (i.e., Chevallard, 1985) and its recent application to e-learning environments directly respond to the question (i.e., Albano, 2017).

Chevallard (1985) introduced a didactical system by raising a question, why mathematics appears in classrooms as it does. His philosophical ground is an anthropologist approach to education in that an epistemological concern (i.e., “what is mathematics and what does it mean to do mathematics”) generates a pedagogical concern (i.e., “how can teachers create a learning environment that enables students to engage with and develop understanding mathematical content?”) (Schoenfeld, 2012, p. 588). Chevallard’s primary interest is on the distance between mathematics practiced outside the classroom and school mathematics to be taught, and he regarded the distance as transformation. His anthropological theory assumes that “mathematics is made of human activities, produced, spread, managed, taught, among a large variety of social institutions” (p. 1). The theory is to justify the distances between academic knowledge produced by mathematicians, knowledge to be taught that is defined by an educational system, knowledge taught by teachers, and knowledge learnt by students (Wozniak et al., 2016, p. 2). Chevallard (1985) believed that the distances build a new concept called, ‘noosphere’ where knowledge to be taught or learnt is set by predetermined social, historical, or cultural contexts (p. 2). This means that the identity changes have already occurred, but we have ignored them.

Albano (2017) applied the didactical system into online learning environments by adding a new vertex, ‘author’ to the top of the didactical triangle vertices, *mathematics-student-tutor*. For Albano, the ‘author’ vertex is necessary to ensure that learners become central in the system. She believed that the educational benefit of online learning environments is that learners become a central role in learning processes, and thus learners should be allowed to plan, develop, and manage the didactic system. To do this, she transformed the didactic triangle into a didactic tetrahedron by adding the author vertex where a learner as an author can be positioned at the three remaining vertices (mathematics-student-tutor) (e.g., learners create resources that modify the milieu used to reach mathematical knowledge; learners officially support other students). For Albano, learners at the author vertex are “both as a single entity and as a community” (p. 354). The author identity is consistent with the high levels of identity changes in this study in that (a) the learners demonstrated and expected to practise their ownership and authorship of learning, (b) the learners and the instructors were co-participants, and (c) the LMS appeared as a learning community. Therefore, it can be reconfirmed that the high levels of identity changes cannot occur unless they are integrated as the foundations for teaching and learning and should reflect the learning environment, like Chevallard aimed to do through his anthropological theory.

Today’s learning environment has been digitally networked, and learning in the network … sustained by the communities as part of the network (Dreamson, 2020). A post-learning community emerges from diverse and dynamic *connective actions* by instructors and learners (Dreamson, 2020). Sun et al. addressed such a challenge in their quantitative survey on learners’ expectations about online communities (n = 740). From the survey analysis, they retrieved three key values including (a) ‘common identity’ — a shared identity is to play an important role in online learning communities, (b) ‘tenuous friendships in online learning communities’ — sociality and bonds emerge from community creation, yet such personal attachments are not critical in an online learning community, and rather, (c) ‘community collective efficacy’ — “identity regulation, coordination and social support account for what online learners as a community are aiming to achieve” could induce more continued engagement (p. 10). In this process, community membership is practised in diverse forms such as individual contributions, group work, sharable reflective activities, group-to-group interactions, and so on. Therefore, a regular motivation check through individual and group consultations should be great encouragement for learners to be more active in online learning communities, which is also the evidence of instructor presence in the community.

**Fig. 1:**
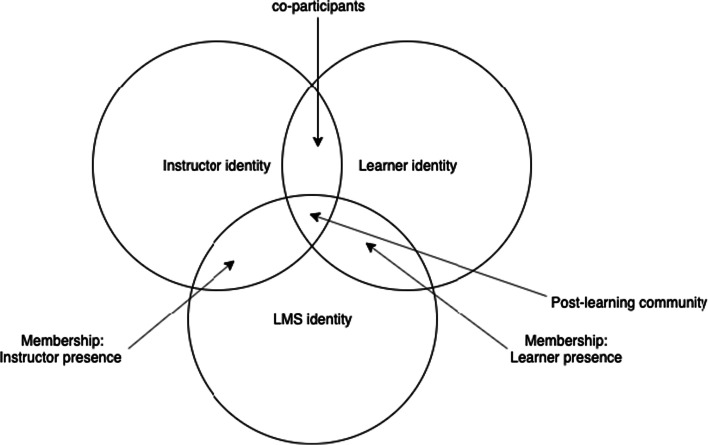
Identity changes

As visualised in Fig. 1, the intersections of the identity changes generate new values for authentic online learning and teaching. Both instructors and learners are supposed to be co-participants who aim to build a quality learning community, and their identity changes occur when the course is moving towards building a post-community by being engaged in their mutual understanding of the identity changes. In this sense, communication and collaboration tools should be structured for active, ongoing, regular contributions by both instructors and learners. This means that an online course is to design and facilitate a learning community on an LMS platform where all the participants are supposed to practise their membership in a collaborative manner towards a post-learning community.

## Conclusion

We conducted this study at a university that had relied on a conventional residential teaching mode and used their LMS as an additive tool. In the contexts, most of the courses remained at the lowest levels of the identity changes. This result implies that (a) diverse levels would be observed when multiple institutes offering mixed teaching modes are included in the sampling, and (b) the quality of identity changes in the three components would be also different in accordance with the types of teaching modes (i.e., online, blended, and flipped modes). Another limitation is highly related to technical, administrative, and pedagogical support for transitioning from offline to online for both instructors and students which is regarded as a determining factor in creating an effective online learning environment (Hart et al., 2021; Kerr-Sims & Baker, 2021). This study does not specifically address such support. In fact, the instructors and the students at the university were given little systematic support due to abrupt transitioning from conventional teaching to online due to the COVID-19. This fact could explain why minimal data for the higher levels of identity changes in this study occurred. Inversely, if systematic support for instructors and students in line with the identity changes, it will be highly possible that the upper levels can be observed.

Even though such limitations should be addressed in future studies, our argument remains meaningful that identity changes can occur when learners and instructors together aim to achieve a post-learning community and ‘their’ LMS is designed to realise it by assuming that online learning and teaching is ongoing development to realise shared values. The intersections of the three components are considered places where new values emerge from their mutual, ongoing engagement that ensure that they are moving towards a post-learning community. Such interactive, relational, and teleological features of online learning and teaching need to be supported at an institutional level. In particular, university leadership should aim to foster a new pedagogical culture for courses to be transformed as post-learning communities by supporting instructors and learners to understand and practice the identity changes. This new culture challenges conventional, fragmented support for individual instructors to obtain technical skills in that all stakeholders should be involved in building a post learning community, as the identity changes are understood as an outcome of the dynamic interactions at an institutional level.

We conclude this study by suggesting the following strategies for authentic online teaching and learning: (a) A learning and teaching division should support instructors to pre-design their online courses by reflecting the identity changes. A course planner for weekly teaching strategies and learning activities can be given to instructors to design their course, and the division gives feedback to instructors for refinement and support them to pre-organise their course sites. (b) Learners need to be ensured that they are expected to be co-participants in learning. In particular, first-year students should be ensured to experience post-learning communities, and therefore, they advocate themselves as authentic co-owners of learning communities in courses throughout the remaining years of their study. This bottom-up approach is expected to bind all stakeholders to authentic online learning and teaching. (c) Post-learning communities need to be recognised, exemplified and shared by all the stakeholders. In practice, course evaluation can be redesigned to reflect to what extent learners and instructors participate in their learning community rather than what individual students perceive the course at a personal level (Park, 2014). Furthermore, course evaluation results can be critically analysed and reflected in the course planner and professional development programs.

## Data Availability

Not applicable.

## References

[CR1] Aboud F (2020). The effect of E: Learning on EFL teacher identity. International Journal of English Research.

[CR2] Albano G, Aldon G, Hitt F, Bazzini L, Gellert U (2017). E-mathematics engineering for effective learning. Mathematics and technology. Advances in mathematics education.

[CR3] Anderson T, Rourke L, Garrison DR, Archer W (2001). Assessing social presence in asynchronous text-based computer conferencing. Journal of Asynchronous Learning Networks.

[CR4] Bonk C, Kirkley J, Hara N, Dennen V, Stephenson J (2018). Finding the instructor in post-secondary online learning: Pedagogical, social, managerial and technological locations. Teaching & learning online: Pedagogies for new technologies.

[CR5] Braun V, Clarke V (2006). Using thematic analysis in psychology. Qualitative Research in Psychology.

[CR6] Brown A, Lawrence J, Basson M, Redmond P (2019). A conceptual framework to enhance student online learning and engagement in higher education. Higher Education Research & Development.

[CR7] Chevallard Y (1985). La transposition didactique—Du savoir savant au savoir enseigné.

[CR8] Conrad, R.M. & Donaldson, J. A. (2012). Transforming the online learner. In: 28th Annual Conference on Distance Teaching & Learning.

[CR9] Conrad, R., & Donaldson, J. A. (2004). *Engaging the online learner: Activities and resources for creative learning*. Jossey-Bass.

[CR10] Cooper T, Scriven R (2017). Communities of inquiry in curriculum approach to online learning: Strengths and limitations in context. Australasian Journal of Educational Technology.

[CR11] Cross J (2004). An informal history of eLearning. On the Horizon.

[CR12] d’Alessio MA, Lundquist LL, Schwartz JJ, Pedone V, Pavia J, Fleck J (2019). Social presence enhances student performance in an online geology course but depends on instructor facilitation. Journal of Geoscience Education.

[CR13] Delahunty J, Verenikina I, Jones P (2014). Socio-emotional connections: Identity, belonging and learning in online interactions: A literature review. Technology, Pedagogy and Education.

[CR14] Dhawan S (2020). Online learning: A panacea in the time of COVID-19 crisis. Journal of Educational Technology Systems.

[CR15] Donaldson, J. A., & Conrad, R. (2005). *Developing learner-led knowledge generating online communities.* In: 20th Annual Conference on Distance Teaching and Learning.

[CR501] Dreamson, N. (2019). *Critical understandings of digital technology in education: Meta-connective pedagogy*. London and New York: Routledge. 10.4324/9780429277528.

[CR502] Dreamson, N. (2020). Online design education: Meta-connective pedagogy. *International Journal of Art & Design Education, 39*(3), 483–497. 10.1111/jade.12314.

[CR503] Dreamson, N., Thomas, G., Lee Hong, A., & Kim, S. (2017). Policies on and practices of cultural inclusivity in learning management systems: perspectives of Indigenous holistic pedagogies. *Higher Education Research and Development, 36*(5), 947–961. 10.1080/07294360.2016.1263830.

[CR500] Felicia, O. (2015). Effect of emotion on distance e-learning: The fear of technology. *International Journal of Social Science and Humanity, 5*(11), 966–970.

[CR16] Harasim L (2000). Shift happens: Online education as a new paradigm in learning. The Internet and Higher Education.

[CR17] Hart CMD, Xu D, Hill M, Alonso E (2021). COVID-19 and community college instructional responses. Online Learning.

[CR18] Heuer BP, King KP (2004). Leading the band: The role of the instructor in online learning for educators. The Journal of Interactive Online Learning.

[CR19] Kerr-Sims S, Baker DMA (2021). Faculty perceptions of teaching online during the COVID-19 university transition of courses to an online format. Journal of Teaching and Learning with Technology.

[CR20] Khalid F (2019). Students’ identities and its relationships with their engagement in an online learning community. International Journal of Emerging Technologies in Learning.

[CR21] Khlaif ZN, Farid S (2018). Transforming learning for the smart learning paradigm: Lessons learned from the Palestinian initiative. Smart Learning Environments.

[CR22] King N, Horrocks C (2010). Interviews in qualitative research.

[CR23] Koole M (2014). Identity and the itinerant online learner. International Review of Research in Open and Distance Learning.

[CR24] Maor D (2003). The teacher's role in developing interaction and reflection in an online learning community. Educational Media International.

[CR25] Maxell JA, Bickman L, Rog DJ (2012). Qualitative research design: An interactive approach. The SAGE handbook of applied social research methods.

[CR26] Palahicky S, DesBiens D, Jeffery K, Webster KS, Keengwe J (2019). Pedagogical values in online and blended learning environments in higher education. Handbook of research on blended learning pedagogies and professional development in higher education.

[CR27] Park JY (2011). Design education online: Learning delivery and evaluation. International Journal of Art & Design Education.

[CR28] Park, J. (2014). Course evaluation: Reconfigurations for learning with learning management systems. *Higher Education Research and Development,**33*(5), 992–1006. 10.1080/07294360.2014.890564

[CR29] Parra JL, Wankel C, Blessinger P (2013). Developing technology and collaborative group work skills: Supporting student and group success in online and blended courses. Increasing student engagement and retention in e-learning environments: Web 2.0. and blended learning technologies.

[CR30] Petticrew M, Roberts H (2006). Systematic reviews in the social sciences: A practical guide.

[CR31] Picciano AG (2017). Theories and frameworks for online education: Seeking an integrated model. Online Learning.

[CR32] Pishva, D., Nishantha, G.G.D., & Dang, H.A. (2010). A survey on how Blackboard is assisting educational institutions around the world and the future trends. In: *2010 The 12th International Conference on Advanced Communication Technology (ICACT)* (pp. 1539–1543). Gangwon, Korea (South): IEEE.

[CR33] Rapanta C, Botturi L, Goodyear P, Guàrdia L, Koole M (2020). Online university teaching during and after the Covid-19 crisis: Refocusing teacher presence and learning activity. Postdigital Science and Education.

[CR34] Reeves S, Albert M, Kuper A, Hodges BD (2008). Why use theories in qualitative research?. BMJ.

[CR35] Richardson JC, Alsup J (2015). From the classroom to the keyboard: How seven teachers created their online teacher identities. International Review of Research in Open and Distributed Learning.

[CR36] Salmon G (2013). E-tivities: The key to active online learning.

[CR37] Santiago BJ, Ramírez JMO, Rodríguez-Reséndiz J, Dector A, García RG, González-Durán JEE, Sánchez FF (2020). Learning management system-based evaluation to determine academic efficiency performance. Sustainability.

[CR38] Schoenfeld AH (2012). Problematizing the didactic triangle. ZDM Mathematics Education.

[CR39] Siemens, G. (2004). *Connectivism: A learning theory for the digital age*. Retrieved from http://www.elearnspace.org/Articles/connectivism.htm

[CR40] Sun N., Rosson, M.B., Carooll, J.M. (2018). Where is community among online learners? Identity, Efficacy and Personal Ties. In: *Proceedings of the 36th annual ACM conference on Human factors in computing systems* (CHI 2018) (pp. 1–13). Montreal, Canada: ACM.

[CR41] Temdee P, López-Ruiz R (2020). Smart learning environment: Paradigm shift for online learning. Multi agent systems - Strategies and applications.

[CR42] Wozniak, F., Bosch, M., & Artaud, M. (2016). Yves Chevallard (English): The anthropological theory of the didactic (pp. 1–7). Retrieved from http://www.ardm.eu/book/export/html/723

[CR43] Wright P (2014). “E-tivities from the front line”: A community of inquiry case study analysis of educators’ blog posts on the topic of designing and delivering online learning. Education in Science.

